# Septin Polymerization Slows Synaptic Vesicle Recycling in Motor Nerve Endings

**DOI:** 10.32607/20758251-2019-11-2-54-62

**Published:** 2019

**Authors:** P. N. Grigoryev, G. A. Khisamieva, A. L. Zefirov

**Affiliations:** Kazan State Medical University, Butlerova Str. 49, Kazan, 420012, Russia

**Keywords:** motor nerve ending, neurotransmitter release, synaptic vesicle cycle, septins, forchlorfenuron

## Abstract

Septins are GTP-binding proteins recognized as a component of the cytoskeleton. Despite the fact that septins are highly expressed by neurons and can interact with the proteins that participate in synaptic vesicle exocytosis and endocytosis, the role of septins in synaptic transmission and the synaptic vesicle recycling mechanisms is poorly understood. In this study, neurotransmitter release and synaptic vesicle exocytosis and endocytosis were investigated by microelectrode intracellular recording of end-plate potentials and fluorescent confocal microscopy in mouse diaphragm motor nerve endings during septin polymerization induced by forchlorfenuron application. It was shown that forchlorfenuron application reduces neurotransmission during prolonged high-frequency (20 and 50 pulses/s) stimulation. Application of pairs of short high-frequency stimulation trains showed that forchlorfenuron slows the replenishment of the readily releasable pool. Forchlorfenuron enhanced FM 1-43 fluorescent dye loading by synaptic vesicle endocytosis but decreased dye unloading from the preliminarily stained nerve endings by synaptic vesicle exocytosis. It was concluded that the septin polymerization induced by forchlorfenuron application slows the rate of synaptic vesicle recycling in motor nerve endings due to the impairment of synaptic vesicle transport.

## INTRODUCTION

Neurotransmitters are secreted in a chemical synapse via exocytosis during the fusion between the membrane of a synaptic vesicle loaded with a neurotransmitter and the presynaptic membrane. This process takes place within specialized structures (active zones) upon opening of Ca^2+^ channels of the presynaptic membrane. A portion of the neurotransmitter released during exocytosis of an individual synaptic vesicle is known as a quantum. The reserve of synaptic vesicles is depleted during neurotransmitter release and replenished during endocytosis and vesicular transport. The presynaptic membrane gives rise to new vesicles, which are loaded with a neurotransmitter, delivered to the active zones, and again used in secretion (the recycling mechanism). A combination of exocytosis, endocytosis, and synaptic vesicle transport constitutes the synaptic vesicle cycle, an important presynaptic mechanism that ensures efficient long-term neurotransmitter release by a neuron. Synaptic vesicles in motor nerve endings are known to be functionally diverse and form several vesicle pools. The readily releasable pool consists of vesicles located in close proximity to the active zone. This pool is limited in size and depletes rather rapidly. It is efficiently replenished by synaptic vesicles from the recycling pool, which are formed from the presynaptic membrane via endocytosis (the short recycling pathway). Synaptic vesicles that constitute the large reserve pool and are formed on the surface of nerve terminal endosomes may be involved in secretion upon prolonged high-frequency neural activity (the long recycling pathway) [1–3]. The mechanisms regulating the presynaptic vesicle cycle and vesicular transport are of great interest to researchers. The cytoskeleton comprising several dynamically polarizing/ depolarizing components (actin filaments, intermediate filaments, microtubules, and septins) can be one of these mechanisms.

Septins are the least-studied cytoskeletal component and belong to the recently discovered conserved family of GTP-binding proteins [4]. Septins are involved in cellular processes, such as cell division, reorganization of other cytoskeletal components, and intracellular transport. By acting as a specific barrier, septins can separate specialized membrane regions from each other [5]. Thirteen septin types (denoted as SEPT1–SEPT12, SEPT14) are known in mammals [6]. They bind to each other to form heterooligomeric complexes that can be polymerized into more complex structures (filaments, rings, and networks). SEPT3, SEPT5–7, and SEPT11 have been identified in mature nerve terminals [7]; however, their functions have not been studied sufficiently. Hence, SEPT5 and SEPT7 are involved in axonal [7] and dendritic [8, 9] growth. SEPT5, SEPT6, and SEPT3 were found to colocalize with synaptic vesicles [7, 10, 11]. It has also been demonstrated that septins interact with a number of the proteins involved in exocytosis: Munc-18-1, synapsin II, VAMP2, synaptophysin, synaptotagmin 1, NSF, Hsc70, etc. It has been suggested that dynamic reorganization of septins is needed for synaptic vesicle exocytosis and neurotransmitter release [12–15]. Even less is known about the role played by septins in endocytosis and synaptic vesicle transport. The interaction of septins with the proteins taking part in endocytosis (clathrin, flotillin, and dynamin) [12, 16] and colocalization of septins and the cell membrane regions rich in phosphoinositol-4,5-bisphosphate, which are required for clathrin-dependent endocytosis [17, 18], imply that septins may be possibly involved in these processes.

Synaptic vesicle recycling upon stimulation of septin polymerization by forchlorfenuron (FCF) was evaluated by using a combination of the electrophysiological approach and confocal fluorescence microscopy. FCF selectively stimulates septin polymerization without affecting other cytoskeletal components (microtubules and actin filaments) and exhibits no cytotoxicity at concentrations up to 500 μM [19].

## EXPERIMENTAL

**Study object and solutions**

Our experiments were performed using isolated neuromuscular specimens of mouse diaphragm. This study was conducted in compliance with the international guidelines for animal experiments. After isolation, the specimen was placed into a recording chamber and subjected to continuous perfusion using a solution for homeotherms with the following composition: NaCl, 125.0 mM; KCl, 2.5 mM; NaH_2_PO_4_, 1 mM; CaCl_2_, 2 mM; MgCl_2_, 1 mM; glucose, 11 mM; and NaHCO_3_, 12 mM. The temperature and pH were maintained at a level of 24°C and 7.3–7.4, respectively. The perfusion solution was continuously saturated with carbogen (95%O_2_/5%CO_2_). All the studies were performed only for the synapses located superficially. The motor nerve was stimulated with suprathreshold 0.2–0.3 ms rectangular pulses; the frequency of pulse trains was 0.2 pulses/s (low-frequency stimulation) or 20 and 50 pulses/s (high-frequency stimulation). Specimen contraction was suppressed using μ-conotoxin GIIIB (Peptide Institute, Inc, Japan) at a concentration of 1–2 μM. Septin polymerization was stimulated by adding forchlorfenuron (50 μM) to the perfusion solution for 40 min. All the substances except for μ-conotoxin GIIIB were purchased from Merch (Germany).

**Electrophysiology**

Single-quantum miniature end-plate potentials (MEPPs) spontaneously arising at rest and multi-quantum end-plate potentials (EPPs) arising in response to motor nerve stimulation were recorded using glass microelectrodes (tip diameter < 1 μm; resistance, 8–10 MΩ) filled with a 2.5 M KCl solution. A microelectrode was inserted into the muscle fiber close to the nerve ending under visual control. The resting membrane potential was controlled using a millivoltmeter. The experiments where changes in the resting membrane potential were > 5 mV were not taken into account. The signals were digitized using a La-2USB A-to-D card. Before applying high-frequency stimulation, 35–100 MEPPs and 7–10 EPPs under low-frequency stimulation were recorded. In order to analyze the number of quanta of the neurotransmitter released in response to each stimulus (the quantal content of EPPs), the amplitude of each recorded EPP and MEPP was normalized to a membrane potential level of -75 mV. The quantal content was calculated as a ratio between the EPP amplitude and the average MEPP amplitude, using correction for nonlinear summation [20, 21].

**Fluorescence microscopy**

Synaptic vesicle exocytosis and endocytosis were studied using a FM 1-43 fluorescent dye (SynaptoGreen C4, Merch) at a concentration of 6 μM. The dye reversibly binds to the presynaptic membrane and is entrapped by the newly emerging synaptic vesicles (is “loaded” into nerve endings) during endocytosis (after stimulation of exocytosis) [22, 23]. In this case, the bright fluorescence observed in the nerve ending demonstrated that the dye was captured by the synaptic vesicles that had undergone exocytosis and endocytosis [23]. Stimulation of exocytosis of pre-loaded vesicles caused the release (“unloading”) of the dye from the nerve endings. Fluorescence was observed using a BX51W1 motorized microscope (Olympus, Germany) equipped with a DSU confocal scanning disc, a CoolLed pE-1 light-emitting diode lamp (CoolLed, UK), and an OrcaR2 CCD camera (Hamamatsu, Japan) connected to a PC using specialized Olympus Cell^P software. The optical equipment used to analyze the fluorescence of FM 1-43 consisted of a set of Olympus U-MNB2 light filters and an Olympus LUMPLFL60xw water immersion objective (1.0 NA). Fluorescence intensity was evaluated using the ImagePro software in arbitrary units (a.u.) as the average fluorescence of pixels in the image of a nerve ending minus the background fluorescence. The background fluorescence was determined as the average fluorescence intensity in a square 50 pixels wide in the image region containing no nerve endings [24].

Statistical data analysis was performed using the Origin software (Origin Lab Corp.). The quantitative results of the study are shown as the mean ± standard error; *n *is the number of independent experiments. Statistical significance was estimated by ANOVA.

## RESULTS

**Neurotransmitter release during prolonged high-frequency stimulation in the presence of forchlorfenuron**

It was established that a 40-min exposure to FCF caused no significant changes in the resting membrane potential of muscle fibers (-73.0 ± 2.8 mV, *n *= 20 and -71.7 ± 3.4, *n *= 20 in the control and test specimens; *p *> 0.05)*. *Low-frequency stimulation resulted in a statistically insignificant reduction in the quantal content of EPPs (59.0 ± 5.8 quanta (*n *= 18) and 53.3 ± 4.7 quanta (*n *= 15) in the control and test specimens, respectively (*p *> 0.05)).

**Fig. 1:**
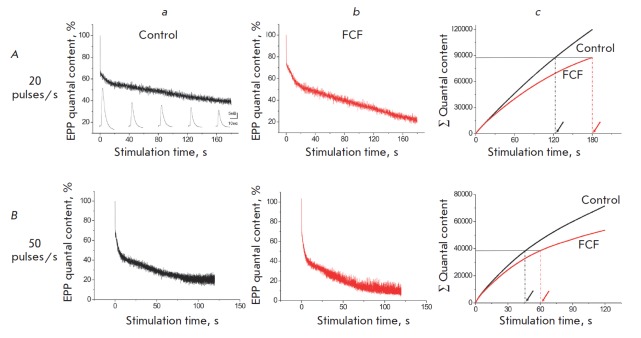
Effect of forchlorfenuron on neurotransmitter release during high-frequency stimulation: *A *– the dynamics of EPP quantal content during prolonged high-frequency stimulation (20 pulses/s) in the control (a) and during FCF application (b). The initial quantal content was taken as 100 %. The averaged experimental data are presented (see the Results section). B – similar curves are shown for 50 pulses/s stimulation. *Ac *and *Bc *are the cumulative curves of released neurotransmitter quanta during high-frequency stimulation. Dotted lines indicate the stimulation time during which the same numbers of quanta are released in the control and during FCF application

Prolonged (3-min) high-frequency stimulation of the control specimen at 20 pulses/s caused a three-phase reduction (depression) in the quantal content of EPP (*Fig. 1Aa*). An initial rapid decline down to 64.7 ± 4.1% (*n *= 9) of the baseline was observed during stimulation for 0.4 s. After a short plateau region lasting approximately 1.5–2 s, there was a second phase corresponding to a slow decline down to 54.2 ± 5.5% (*n *= 9) of the baseline by 15 s of stimulation. A further, even slower, decline reduced the quantal content of EPP down to 35.9 ± 6.5% (*n *= 9) of the baseline by 3 min of stimulation. Stimulation of the motor nerve at a higher frequency (50 pulses/s) for 2 min yielded similar three-phase dynamics of quantal content reduction, but depression of the neurotransmitter release was more pronounced (*Fig. 1Ba*). The initial rapid decline reaching 72.3 ± 3.0% (*n *= 9) of the baseline was no longer observed by approximately 0.16 s of stimulation. After the short plateau phase lasting 0.5–0.8 s, there followed a second reduction phase, which was characterized by slower decline kinetics. The third, even slower, phase started on the 5^th^ second of stimulation. By the end of the 2^md^ minute of stimulation, the quantal content was at 22.7 ± 7.0% (*n *= 9) of the baseline.

No qualitative changes in the dynamics of decline in the quantal content of EPP were observed in the presence of FCF (there was a three-phase decline); however, the depression magnitude was deeper. During high-frequency stimulation (20 pulses/s, *Fig. 1Ab*), the quantal content of EPP decreased, down to 20.2 ± 6.8% (*n *= 7) of the baseline by the 3^rd^ minute of stimulation. Upon stimulation at a frequency of 50 pulses/s, the decline in the quantal content of EPP was also more pronounced (*Fig. 1Bb*). By the end of the 2^md^ minute of stimulation, the quantal content in the test specimens had dropped down to 6.2 ± 3.6% (*n *= 9) of the baseline.

The cumulative curves showing the number of released neurotransmitter quanta revealed that the intensity of neurotransmitter release was statistically significantly reduced in the presence of FCF. Three-minute stimulation at a frequency of 20 pulses/s led to a release of 119,796 ± 8,161 quanta (*n *= 9) in the control specimens, while the neurotransmission after application of FCF was down by 27% (87,611 ± 9,025 quanta (*n *= 7), *p* < 0.05 (*Fig. 1Ac*). The two-minute stimulation at a frequency of 50 pulses/s resulted in a release of 71,505 ± 5,543 quanta (*n *= 9) in the control specimen; neurotransmission in the presence of FCF was lower by 25%: 53,553 ± 8,904 quanta (*n *= 9), *p* < 0.05 (*Fig. 1Bc*).

The deepened depression of neurotransmitter release upon high-frequency stimulation in the presence of FCF can be attributed to a suppression of mobilization (the replenishment rate of the readily releasable pool).

**The replenishment rate of the readily releasable pool upon high-frequency stimulation in the presence of forchlorfenuron**

**Fig. 2:**
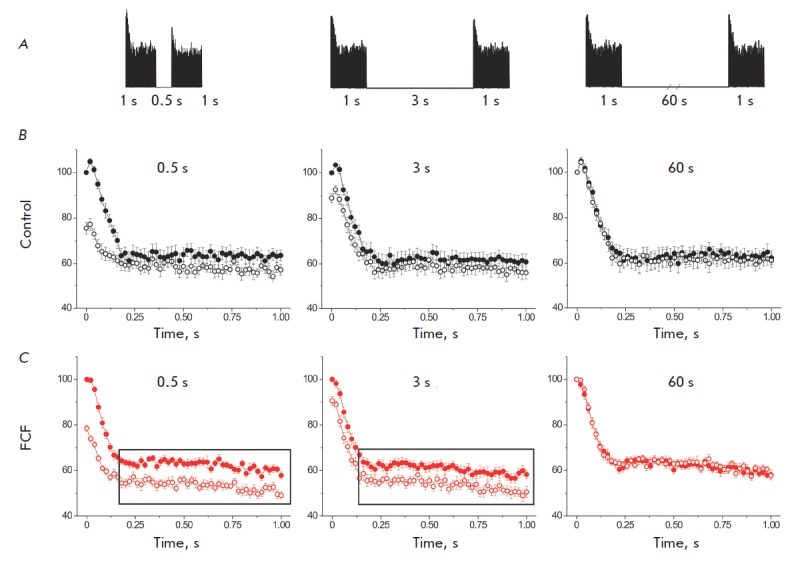
Effect of forchlorfenuron on the replenishment of the readily releasable pool during high-frequency stimulation. *A *– The experimental scheme. Pairs of short (1 s) stimulation trains with a frequency of 50 Hz and a delay time of 0.5, 3 and 60 s between the first and the second train were given. *B *and *C *– The dynamics of neurotransmitter release during the first (dark circles) and the second (white circles) trains in the control and during FCF action. In each experiment, the value of the quantal content of the first EPP in the first train was taken as 100 %. It is noticeable that application of FCF leads to stronger depression of neurotransmitter release during the second stimulation train at delay times of 0.5 and 3 s than in the control experiments

In order to assess the replenishment rate of the readily releasable pool, we applied short (1 s) pulse trains at a frequency of 50 pulses/s, with different intervals between the trains (0.5, 3, and 60 s), to normal specimens and specimens in the presence of FCF [25]. In response to the first pulse train, the quantal content of EPP decreased abruptly during the first 6–8 pulses. Next, there followed a plateau (the quantal content remained at the same level) (*Fig. 2B,C*). Summation of the number of released quanta demonstrated that they were identical both in the control specimen and in the specimen exposed to FCF after the first pulse train (2,214 ± 192 (*n *= 12) and 2,205 ± 194 quanta (*n *= 12); *p *> 0.05). Therefore, the entire readily releasable pool (being ~1,700 quanta in mouse motor nerve terminals) was involved in secretion after the first pulse train [25, 26], while virtually not affecting the recycling pool (~ 80,000 quanta) [27]. A lower secretion level was observed for the second pulse train applied 0.5 and 3.0 s after the first one (*Fig. 2 B,C*) because of the incomplete replenishment of the readily releasable pool. Thus, the total number of quanta released during the second pulse train in the control specimen and in the specimen exposed to FCF was 88.3 ± 1.0% (*n *= 12) and 83.9 ± 1.0% (*n *= 12), respectively, of the number of quanta released during the first pulse train (*p* < 0.01). When the interval between the pulse trains stood at 3 s, the number of quanta released was 93.0 ± 0.8% (*n *= 13) and 88.5 ± 1.2%, respectively (*n *= 13); *p* < 0.01. Therefore, neurotransmitter release in the specimen exposed to FCF was recovered much less efficiently than that in the control sample; the most significant changes were observed during the plateau phase (*Fig. 2 B,C*). At high intervals between the pulse trains (60 s), secretion recovered completely: to 100.1 ± 1.0% (*n *= 12)) in the control specimens and 98.9 ± 0.7% (*n *= 12) in the specimens exposed to FCF. Taking into account that the average time of synaptic vesicle recycling in mouse motor nerve terminals is ~ 50 s [27], it is fair to assume that replenishment of the readily available pool during a short interval between pulse trains (0.5 and 3 s) occurs due to the recycling pool only, but not due to synaptic vesicle endocytosis. Therefore, application of FCF does not alter the readily releasable pool but slows its replenishment rate due to the recycling pool.

**FM 1-43 loading into nerve terminals in the presence of forchlorfenuron**

**Fig. 3:**
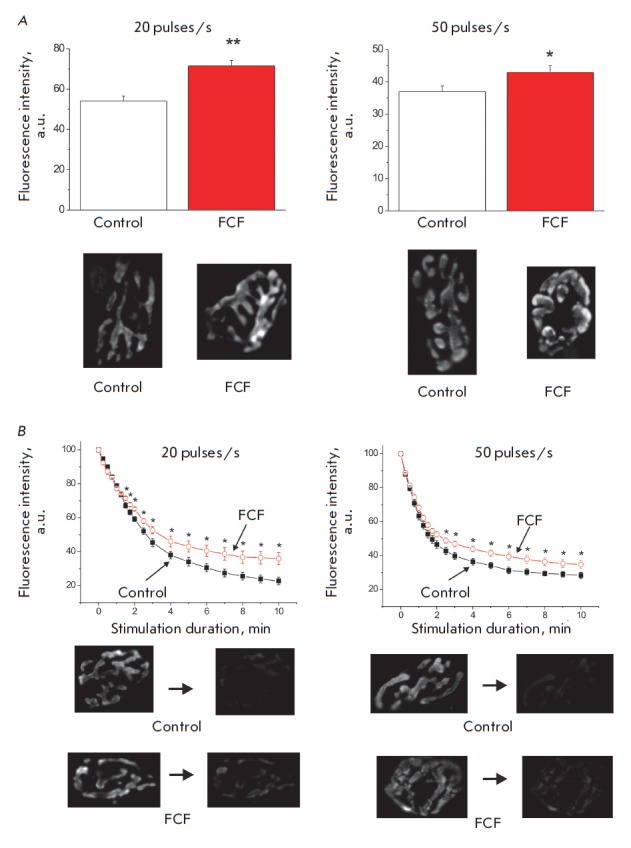
Effect of forchlorfenuron on FM 1-43 loading and unloading during high-frequency stimulation. *A *– Fluorescence intensity of nerve endings loaded with FM 1-43 at high-frequency stimulation (20 and 50 pulses/s) in the control and during FCF application in case of equal neurotransmitter release (described in details in the Results section). The fluorescence images of nerve endings from individual experiments are shown below. *B *– The dynamics of fluorescence intensity decay (dye unloading) of preliminarily stained nerve endings in the control (black squares) and upon FCF action (white circles) during high-frequency stimulation (20 and 50 pulses/s). In each experiment, the initial nerve ending fluorescence was taken as 100 %. Fluorescence images of nerve endings from individual experiments before and at the end of stimulation are shown below

Aggravation of suppression of neurotransmitter release in response to prolonged high-frequency stimulation in the presence of forchlorfenuron (*Fig. 1*) can also be related to the disturbed synaptic vesicle endocytosis. We have attempted to test this assumption in experiments using the FM 1-43 fluorescent dye. It is known that the processes of synaptic vesicle endocytosis follow exocytosis at a 1 : 1 ratio. Therefore, special experimental conditions need to be created to evaluate endocytosis under which the levels of neurotransmitter release are identical in the control and test (FCF application) series and identical numbers of vesicles undergo exocytosis. An analysis of the cumulative curves of neurotransmitter release (*Fig. 1Ac*) demonstrated that the numbers of neurotransmitter quanta released in response to a 2- and 3-min stimulation of the control and test specimens at a frequency of 20 pulses/s were almost identical. These stimulation times were used to study FM 1-43 loading. If the endocytosis processes are not affected, one can expect the degree of dye loading and fluorescence intensity to be identical. However, the fluorescence intensity in the nerve terminals in the presence of FCF was much higher than that in the control specimens: 71.6 ± 2.7 a.u. (*n *= 123) and 54.1 ± 2.3 a.u. (*n *= 123), respectively; *p* < 0.01 (*Fig. 3A*). Stimulation at a higher frequency (50 pulses/s) lasting 45 s in the control series and 1 min in the test series (the number of released quanta are also equal under these conditions (*Fig. 1Bc*)) resulted in a statistically higher fluorescence intensity in the specimens exposed to FCF (42.9 ± 2.1 a.u. ((*n *= 125)) compared to that in the control specimens (37.0 ± 1.7 a.u. (*n *= 125); *p* < 0.05) (*Fig. 3A*). Therefore, stimulation of septin polymerization by FCF increased the number of dye-loaded vesicles in the nerve terminal.

**Unloading FM 1-43 from the nerve terminals in the presence of forchlorfenuron**

In this experimental series, we evaluated the effect of FCF on synaptic vesicle exocytosis. At the first stage, all the specimens were loaded with FM 1-43. For this purpose, we applied a prolonged 3-min stimulation of the motor nerve at a frequency of 20 pulses/s in a solution containing FM 1-43 [28]. The control specimens were then perfused with a standard solution, while the test specimens were perfused with a solution containing FCF. Prolonged high-frequency stimulation was applied 40 min later (*Fig. 3B*). The fluorescence intensity of the nerve terminal decayed efficiently as In this experimental series, we evaluated the effect of FCF on synaptic vesicle exocytosis. At the first stage, all the specimens were loaded with FM 1-43. For this purpose, we applied a prolonged 3-min stimulation of the motor nerve at a frequency of 20 pulses/s in a solution containing FM 1-43 [28]. The control specimens were then perfused with a standard solution, while the test specimens were perfused with a solution containing FCF. Prolonged high-frequency stimulation was applied 40 min later (*Fig. 3B*). The fluorescence intensity of the nerve terminal decayed efficiently as

## DISCUSSION

Like other cytoskeletal components, septins are present in the cell in the polymerized and depolymerized forms. Polymerized septins form near the cytoplasmic membrane [29]; therefore, one can expect them to be directly involved in the regulation of the processes of the synaptic vesicle cycle taking place near the presynaptic membrane. Application of forchlorfenuron is one of the most potent and convenient tools that can be used to study the septin function. The effects of FCF and other methods used to impair septin function (application of small interfering RNA and transgenic animals) were shown to be identical in studies focused on various cellular mechanisms [12, 30–34].

**Septins in neurotransmitter release and synaptic vesicle exocytosis**

The role played by septins in the regulation of exocytosis and neurotransmitter release is rather controversial. Thus, it had been assumed that SEPT8 facilitates exocytosis by separating the complex of vesicular proteins VAMP2/synaptophysin. These proteins further interact with SNAP25, and the SNARE complex assembly takes place [14]. Meanwhile, it was discovered that polymerized SEPT5 can form a physical barrier within the active zones, which hinders synaptic vesicle exocytosis and can affect the distance between a calcium channel and a synaptic vesicle [10]. Knockout mice unable to express SEPT4 exhibited lower levels of neurotransmitter release [35]. The disturbed function of the ubiquitously expressed septin variant SEPT2 revealed that exocytosis was altered [12]. Meanwhile, no significant alterations in neurotransmitter release were detected in mice unable to express SEPT5 and SEPT3 [36]. It was demonstrated that stimulation of septin polymerization by FCF reduces the intensities of synchronous, asynchronous, and spontaneous neurotransmitter release in mouse motor neurons [12]. We also detected that application of FCF causes a less pronounced but equivocal decrease in the quantal content. These differences are possibly related to the fact that different durations of exposure to FCF and non-identical extracellular calcium concentrations were used. It is known that higher concentrations or exposure durations potentiate the effect of forchlorfenuron [19]. We used an exposure duration of 40 min, while Tokhtaeva et al. [12] applied a longer exposure duration (1 h). This could be the reason why we observed a weaker effect of stimulation of septin polymerization on the function of SNARE proteins.

**Septins and synaptic vesicle endocytosis**

The intensity of fluorescent dye loading and fluorescence of nerve endings after exposure to FCF was much higher than that in the control specimens, while the intensities of neurotransmitter secretion were identical (and exocytosis was identical as well) (*Fig. 3A*). In other words, the number of stained vesicles per nerve ending was greater. These data could have been interpreted as enhancement of synaptic vesicle endocytosis. However, this conclusion can be drawn only if vesicles loaded with the dye were repeatedly engaged in neurotransmitter release at the same rate. An analysis of the curves of dye unloading (*Fig. 3B*) demonstrated that stimulation at frequencies of 20 and 50 pulses/s reduced the rate of release of the pre-loaded FM 1-43 fluorescent dye. This fact indicates that delivery of dye-loaded vesicles to the secretion sites has been slowed down rather than that endocytosis has been enhanced. Meanwhile, the divergence between the curves of unloading dynamics during the first several minutes of recording was not sufficiently significant to be able to independently cause a reduction in the number of released neurotransmitter quanta of 25–27% in the presence of FCF. Therefore, disruption of transport and recycling of the synaptic vesicles formed by endocytosis immediately during high-frequency stimulation can be regarded as another explanation to the reduction in the neurotransmitter release upon septin polymerization. This can also be supported by the revealed enhanced loading of the FM 1-43 fluorescent dye (*Fig. 3A*). Thus, in the control specimens, a vesicle population is repeatedly involved in exocytosis and releases FM 1-43, along with the neurotransmitter, while the number of synaptic vesicles carrying the dye in a nerve ending becomes lower than the expected value after high-frequency stimulation. Exposure to FCF reduces the percentage of vesicles repeatedly involved in secretion because of the impaired transport of synaptic vesicles that newly emerges during endocytosis. As a result, the number of synaptic vesicles loaded with the fluorescent dye in the presence of FCF is higher than that in the control, which is the reason why the FM 1-43 fluorescence in the presence of FCF is brighter (*Fig. 3A*). Meanwhile, exposure to FCF can also impair the endocytosis mechanism and the entire process can be slowed down. The histochemical data demonstrating that SEPT3 is substantially colocalized with dynamin indicate that septins can potentially be involved in synaptic vesicle endocytosis [37]. Other septins, SEPT5 and SEPT9, were also found to interact with dynamin [16].

**Septins and synaptic vesicle transport**

In the beginning of prolonged high-frequency stimulation, vesicles from the readily releasable and recycling pools take part in the neurotransmitter release. Later (after several dozen seconds), the newly emerging vesicles can be repeatedly involved in secretion. To ensure this, a new synaptic vesicle needs to form via endocytosis, be loaded with a neurotransmitter, get into the recycling pool, and subsequently replenish the readily releasable pool via mobilization. In other words, the vesicle transport route consists of two components: the recycling pool - readily releasable pool and the endocytosis - recycling pool. In all likelihood, septins ensure the functioning of both components. The electrophysiological data demonstrating a reduced replenishment rate of the readily releasable pool after high-frequency stimulation indicate that vesicle transport to the active zones is less efficient upon septin polymerization (*Fig. 2*). The data on enhanced loading and slowed-down unloading of FM 1-43 indicate that vesicle transport from the endocytosis sites is suppressed (*Fig. 3 A,B*).

Involvement of septins in the functioning of the actomyosin motor can be considered a mechanism through which they participate in synaptic vesicle transport. It was established that septins can interact with actin [38] and nonmuscle myosin II [39], which take part in synaptic vesicle transport [40–42]. The ability of polymerized septins to form barriers impeding synaptic vesicle transport is one of the mechanisms that explain why the intracellular transport is impaired [5, 10].

## CONCLUSIONS

Hence, our findings demonstrate that septins are involved in the processes of synaptic vesicle cycle and synaptic vesicles reuse in neurotransmitter release during prolonged high-frequency activity of a neuromuscular junction.

## Abbreviations

EPP: end-plate potential
FCF: forchlorfenuron
